# Evaluation of Marginal Adaptation of SDR Plus, Fiber-Reinforced and Nanofilled Composites in Endodontically Treated Teeth: A Scanning Electron Microscopic Study

**DOI:** 10.7759/cureus.70745

**Published:** 2024-10-03

**Authors:** Gagandeep Kaur, Rajinder K Bansal, Manu Bansal, Dolphi Bansal, Reeshu Garg, Sakshi Singla, Seema Gupta

**Affiliations:** 1 Department of Conservative Dentistry and Endodontics, Guru Nanak Dev Dental College and Research Institute, Sunam, IND; 2 Department of Orthodontics, Kothiwal Dental College and Research Centre, Moradabad, IND

**Keywords:** composite resins, in-vitro study, marginal adaptation, microleakage, scanning electron microscopy

## Abstract

Introduction: Endodontically treated teeth (ETT) undergo structural changes, including a reduction in water content and loss of dentin elasticity, which can compromise their mechanical properties. One critical factor influencing the long-term prognosis of a restored ETT is the marginal adaptation of the restorative material. The present study compared the marginal adaptability of Smart Dentin Replacement (SDR) Plus, fiber-reinforced, and nanofilled composites in ETT using scanning electron microscopy (SEM).

Materials and methods: Cavities were made in 30 recently extracted maxillary premolars distributed into three experimental groups depending on the restorative material used: group 1 as SDR Plus (Dentsply Sirona, Charlotte, North Carolina, USA), group 2 as fiber-reinforced composite (EverX, GC Corp., Tokyo, Japan), and supra nanofilled composite (Estelite Sigma Quick, Tokuyama Dental Corp., Tokyo, Japan). Standardized access openings were performed, and endodontic treatment was completed in all teeth. Specific composite restoration techniques were applied according to the manufacturer’s instructions. After thermocycling for 30 days, the restored teeth were sectioned and viewed using SEM (Labindia Instruments Pvt. Ltd., Maharashtra, India) to measure the width of the marginal gap. The data were analyzed using statistical software.

Results: The mean marginal gap width varied significantly among the groups. Group 1 had a lower mean marginal gap of 2.99 μm, group 2 exhibited the highest mean marginal gap of 10.24 μm, and group 3 had a mean marginal gap of 5.51 μm. Overall, group 2 consistently showed significantly larger marginal gaps than the other groups.

Conclusion: Within the limitations of this study, SDR Plus demonstrated better marginal adaptation than fiber-reinforced and supra-nanofilled resins in the coronal restoration of ETT. Fiber-reinforced composites demonstrated the maximum marginal gap, followed by nanofilled composites. Thus, SDR Plus may be recommended as a suitable restorative material to enhance the longevity and success of endodontic treatments.

## Introduction

Recent investigations in the field of endodontics have outlined the critical importance of coronal microleakage in determining the long-term efficacy of teeth that have undergone endodontic treatment. Coronal leakage has the potential to function as a reservoir for both microorganisms and nutrients, thereby initiating and perpetuating periradicular inflammation. This phenomenon may serve as a principal factor contributing to the failure of endodontic procedures [1]. Notably, a root canal system that is meticulously obturated yet lacks adequate coronal sealing can facilitate the ingress of microorganisms into the periapical tissues [1,2]. Consequently, the mere presence of a well-obturated root canal does not suffice as an effective barrier against leakage. Implementing proper marginal sealing through coronal restoration is imperative for preserving apical periodontal health in teeth subjected to endodontic treatment [2,3].

Endodontically treated teeth (ETT) experience significant structural alterations and loss of tooth substance, notably a decrease in moisture content and a reduction in the elasticity of the dentin, which can adversely affect their mechanical properties [4]. Consequently, the efficacy of restorative interventions in ETT predominantly hinges upon the quality of the employed materials and their capacity to form a robust bond with the tooth structure [4,5]. A pivotal determinant impacting the long-term success of a restored ETT is the marginal adaptation of the restorative material. Marginal adaptation is defined as the capacity of a restorative substance to closely adhere to the cavity walls while minimizing the presence of gaps at the interface between the tooth and the restoration. Inadequate marginal adaptation may result in microleakage, recurrent caries, and, ultimately, the failure of the restoration [5]. Therefore, the attainment of optimal marginal adaptation is essential for ensuring the durability and clinical success of restorations in ETT [6].

The marginal adaptation of restorative materials is paramount in mitigating the prevalent clinical failures frequently associated with ETT restorations. In instances where interfacial gaps are present between the restorative material and the tooth structure, there exists a potential for the ingress of oral fluids, bacteria, and debris, culminating in microleakage [5]. This microleakage is a significant factor in the onset of secondary caries, post-operative sensitivity, tooth discoloration, and restoration failure. Furthermore, ETTs exhibit an increased susceptibility to fracture due to their compromised structural integrity, and insufficient marginal adaptation further exacerbates this vulnerability [6]. Consequently, attaining a hermetic seal is critical for preserving the ETT's structural integrity, augmenting the tooth's longevity, and minimizing the necessity for subsequent retreatment.

A variety of composite materials have been engineered to enhance marginal adaptation, such as bulk-fill composites, which have surged in popularity due to their diminished polymerization shrinkage and their capacity to be applied in more substantial layers, thereby reducing the likelihood of the formation of marginal gaps [6]. Furthermore, flowable composites have demonstrated the potential to enhance marginal adaptation as a result of their reduced viscosity, which facilitates superior adaptation to cavity walls. Nonetheless, flowable composites generally exhibit inferior mechanical strength relative to conventional hybrid or nano-hybrid composites [7]. Investigations have indicated that nano-hybrid composites provide an advantageous equilibrium between mechanical properties and marginal adaptation, rendering them appropriate for the restoration of ETT [7,8]. Contemporary resin composites, including smart dentin replacement (SDR) and supra-nanofilled composites, incorporate polymerization modulators that mitigate polymerization shrinkage [9].

Many methodologies have been utilized to assess the marginal adaptation of restorative materials. These methodologies encompass dye penetration, scanning electron microscopy (SEM), micro-computed tomography (micro-CT), and replication techniques [6,10]. Dye penetration represents one of the more rudimentary approaches; however, it is constrained by its inherently qualitative characteristics. SEM is a prevalent technique employed to analyze the marginal adaptation of restorative materials in teeth subjected to endodontic treatment, owing to its superior imaging resolution and capacity to furnish intricate insights into the interface between the tooth and restoration [11,12]. The current investigation compared the marginal adaptability of SDR Plus (Dentsply Sirona, Charlotte, North Carolina, USA), fiber-reinforced (EverX, GC Corp., Tokyo, Japan), and supra-nanofilled composites (Estelite Sigma Quick, Tokuyama Dental Corp., Tokyo, Japan) in ETT, utilizing SEM as the primary analytical tool. The null hypothesis that was subjected to testing posited that there would be no statistically significant difference in the marginal gap at the interface between the tooth and restoration after aging among the groups.

## Materials and methods

Study setting

This in vitro study was conducted at the Department of Endodontics, Guru Nanak Dev Dental College, Sunam, Punjab, from June 2022 to January 2023. The study was approved by the Institutional Review Board (GNDDC/2021/135) and followed the guidelines of the Declaration of Helsinki 2007.

Sample size estimation

The sample size was estimated by using G Power software version 3.6.9 to ensure an adequate power of 80% with an alpha error of 5% (Heinrich-Heine-Universität Düsseldorf, Düsseldorf, Germany). Based on an effect size of 0.59, which was derived from a previous study [6], a total sample size of 30 (10 per group) was determined to be sufficient. The referenced study investigated the marginal adaptation of different bulk-fill composites and reported a mean difference in the marginal gap of 0.29 μm (ranging from 0.379 to 0.069 μm) between SDR and fiber-reinforced composite materials. The pooled standard deviation was 0.49.

Sample selection

Thirty human maxillary first premolars with fully matured apices that had been recently extracted for orthodontic reasons were collected from the Department of Oral Surgery. Teeth exhibiting indications of prior fillings, decay, unusual crown shapes, signs of cracks, or past root canal treatment were excluded from the study. The teeth were cleaned off for any kind of tissue debris and calculus with scalers and disinfection by dipping in a 5% sodium hypochlorite (NaOCl) solution (Morphisto GmbH, Hessen, Germany). Teeth were preserved in 0.9% normal saline (Albert David Ltd., Kolkata, India) until further investigation.

Methodology

**Figure 1:**
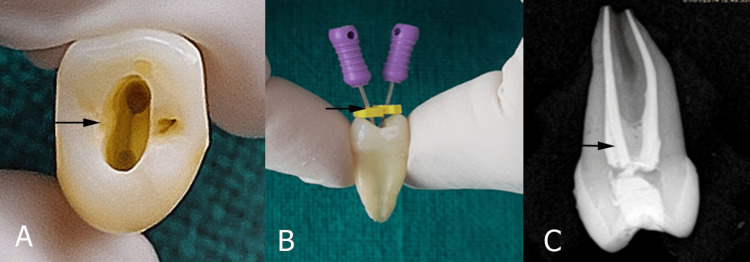
Access opening and obturation. (A) Access opening to the canal, (B) biomechanical preparation, and (C) obturation quality evaluation.

Standard endodontic access openings were made in all 30 samples by using a high-speed handpiece (Dentmark, Ludhiana, India). The access cavity preparation in upper first premolars was done by an experienced operator to reduce variability and had been trained to ensure consistency. A vernier caliper was used to create cavities of consistent size across all samples. The buccolingual dimension of the cavity was 6 mm, and the mesiodistal dimension was 4 mm. The depth of the access cavity was deep enough to expose the pulp chamber (Figure 1A).

Preparation was performed to simulate the access cavity in teeth with class 1 deep dentinal caries with intact marginal ridges. The pulp was de-roofed with a #BR 41 round bur (MANI, Inc., Utsunomiya, Japan), then extended with a non-end cutting bur (MANI, Inc.). Canal length was measured using a no. 10 stainless steel K-file (MANI Inc.) until the tip was observed at the apical foramen (Figure 1B). From the obtained length, 1 mm was subtracted and defined as the final working length.

Biomechanical root canal preparation was performed by preparing a glide path with #15 no., 2% K-file (MANI, Inc.), followed by nickel-titanium (Niti) rotary files. The orifice was enlarged with file # 30 no. 8% (Neo Endo Flex, Orikam, Gurgaon, Haryana, India). The canals were shaped in sequential order (17 no., 4%; 20 no., 4%; 25 no., 4% to the size 35 no., 4%) at a speed of 350 rpm and a torque of 1.5 N.cm. Canals were irrigated alternatively with 5% NaOCl and normal saline during preparation. Final irrigation was performed with a 17% ethylenediaminetetraacetic acid solution (Prevest DenPro, Jammu, India) to dislodge the smear layer, followed by normal saline. After drying the canal with the paper point, a master cone radiograph of each sample with 35 no., 4% gutta-percha point was selected, and obturation of all the teeth was performed using a single cone technique with a zinc oxide-based sealer (ZOE) (Endoseal, Prevest DenPro, Jammu, India). The quality of obturation was evaluated by post-operative radiography (Figure 1C). The reason for choosing ZOE sealer in our study was that the composites, particularly bulk-fill materials, may experience some degree of polymerization shrinkage. ZOE sealer, with its flowable nature and minimal shrinkage, can help compensate for any gaps that might form due to the shrinkage of the composite material. The single cone methodology was employed in our investigation due to its ability to deliver exceptional apical sealing, a major concern in endodontics. Moreover, this method is often simpler and faster than more complex lateral or vertical compaction techniques. This technique can be easily standardized, reducing variability between operators in a study. Temporary coronal sealing was performed with temporary restorative material (Neo Temp, Orikam, Gurgaon, Haryana, India) for seven days to complete the setting of the sealer.

**Figure 2:**
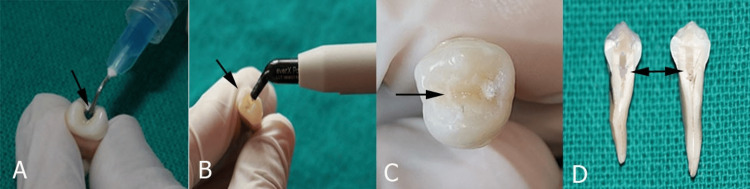
Composite filling. (A) Etching the enamel surface, (B) composite filling and curing, (C) composite surface after thermocycling, and (D) section the tooth longitudinally in mesiodistal direction.

All 30 samples were equally distributed into three groups according to the composite filling chosen. After seven days, the temporary restoration was removed. The cavities were cleaned, and the enamel was etched with 37% phosphoric acid (N-ETCH, Ivoclar, Schaan, Liechtenstein) for 20 seconds (Figure 2A).

The dentin was then rinsed with water for 15 seconds and blot-dried, leaving it moist and shiny. Then, a universal adhesive system (Tetric-N-Bond Universal, Ivoclar, Schaan, Liechtenstein) was applied in two consecutive coats and then light-cured for 20 seconds, as recommended by the manufacturer. Subsequently, the composite restoration was completed (Figure 2B). In group 1, the teeth were restored with bulk-fill composite SDR Plus. In group 2, the teeth were restored with fiber-reinforced composites. In group 3, the teeth were restored with supra-nanofilled composite using a horizontal incremental layering technique.

After post-endodontic restoration, all teeth were subjected to thermocycling (Holmarc Opto-Mechatronics Ltd., Kochi, Kerala) for 30 days with 500 cycles each day at 5-55 °C with a dwell time of 30 seconds and a transfer time of 5 seconds (Figure 2C).

**Figure 3:**
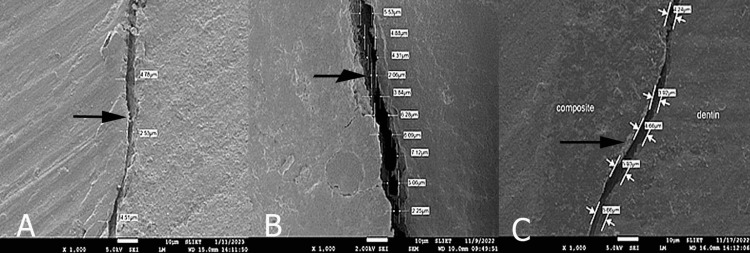
Scanning electron microscopic (SEM) images at 1000× magnification showing marginal gap in micrometer: (A) group 1, (B) group 2, (C) group 3.

Each sample was divided longitudinally in a mesiodistal direction from the center of the filling using a diamond disc (Figure 2D). All sectioned samples were gold-sputtered and viewed using an SEM at 1000× magnification (Labindia Instruments Pvt. Ltd., Maharashtra, India). The gap between the restoration and the tooth surface was measured and expressed in micrometers (Figure 3).

Statistical analysis

The data obtained were entered into Microsoft Excel and analyzed using the Statistical Package for the Social Sciences (SPSS) software (IBM Corp., Released 2013; IBM SPSS Statistics for Windows, Version 22.0. Armonk, NY: IBM Corp.). The data were checked for normal distribution using the Shapiro-Wilk test. Continuous variables were expressed as means and standard deviations. A comparison of the mean values was performed using a two-way analysis of variance (ANOVA). Significance was set at p<0.05, and significant values were analyzed using post-hoc analysis.

## Results

The comprehensive descriptive analysis of the study cohorts (Table 1) indicated notable discrepancies in the mean values pertaining to the marginal gap. Group 2 demonstrated the most pronounced marginal gap, with a mean measurement of 10.25 ± 4.89 µm. Conversely, group 1 exhibited the least mean marginal gap, calculated at 2.99 ± 0.95 µm, with a range extending from 1.7 to 4.28 µm and a 95% confidence interval for the mean spanning from 2.31 to 3.67 µm, thereby implying a more uniform performance. Group 3 exhibited a mean marginal gap quantified at 5.51 ± 2.09 µm. The 95% confidence interval for the mean of group 3 was established between 4.02 and 7 µm.

**Table 1:** Descriptive analysis of study groups comparing marginal gap (in micrometer). Data presented in form of mean ± standard deviation (SD).

Groups	N	Minimum	Maximum	95% Confidence interval for mean	Mean ± SD
Group 1	10	1.7	4.28	2.31–3.67	2.99 ± 0.95
Group 2	10	4.74	18.36	6.75–13.74	10.25 ± 4.89
Group 3	10	3.31	9.09	4.02–7	5.51 ± 2.09

The findings derived from the one-way ANOVA analysis revealed a statistically significant disparity among the groups concerning the marginal gap, evidenced by a p-value of 0.001. The F-ratio of 13.95, determined from the mean square values (135.71 for the groups and 9.73 for the residual), substantiates the dismissal of the null hypothesis, indicating that the variation observed between the groups is not attributable to random variability. The effect size of 1.02 is remarkably substantial, signifying a robust association between the type of composite utilized and the marginal gap. This outcome underscores that the choice of composite material substantially affects the marginal adaptation (Table 2).

**Table 2:** Comparison of the mean marginal gap (micrometer) between the three groups using one-way ANOVA test. *p< 0.05: significant; df: degree of freedom.

	Sum of squares	df	Mean square	F-value	p-value	Effect size
Group	271.42	2	135.71	13.95	0.001*	1.02
Residual	262.67	27	9.73			
Total	534.09	29	-			

The pairwise analysis of the groups utilizing the post-hoc Bonferroni test demonstrates substantial disparities in the marginal gap across specific groups. Group 2 exhibited a notably greater marginal gap in comparison to group 1, with a mean difference quantified at 7.26 μm (p = 0.001). This finding suggests that Group 2 possessed a markedly larger marginal gap than group 1. Furthermore, group 2 also displayed a significantly increased marginal gap relative to group 3, with a mean difference of 4.74 μm (p = 0.006). However, the comparison conducted between group 1 and group 3 did not yield a statistically significant difference (Table 3).

**Table 3:** Pairwise comparison of groups by post-hoc Bonferroni test. *p<0.05: significant; CI: confidence interval.

Pair wise group		Mean difference	Standard error	t-value	p-value	95% CI (lower limit)	95% CI (upper limit)
Group 2	Group 1	7.26	1.395	5.20	0.001*	3.67	10.84
Group 2	Group 3	4.74	1.395	3.39	0.006*	1.15	8.32
Group 1	Group 3	−2.52	1.395	−1.81	0.246	−6.11	1.07

## Discussion

The present study aimed to evaluate and compare the marginal adaptation of SDR Plus, fiber-reinforced composites, and nanofilled composites in ETT using SEM. Based on these results, the null hypothesis, which suggests no significant difference in marginal adaptation between the composite groups, is rejected. Significant differences in the mean marginal gap were observed, with fiber-reinforced composites exhibiting the largest marginal gaps compared with SDR Plus and nanofilled composites. This finding highlights that not all modern composite materials offer superior marginal adaptation, particularly in ETT, where structural integrity is compromised [13].

The marginal gap between the tooth and composite interface is a critical factor that can lead to a range of clinical complications. Poor marginal adaptation allows penetration of oral fluids, bacteria, and debris, leading to microleakage [7,10]. Microleakage is associated with secondary caries, post-operative sensitivity, and discoloration of both the restoration and the tooth [14]. Moreover, in endodontically treated teeth, where the tooth structure is already weakened, larger marginal gaps can increase the risk of fracture, particularly under functional load. The formation of gaps can also compromise the longevity of the restoration, often necessitating replacement or retreatment [15].

In the present study, factors that affect polymerization shrinkage, such as light intensity, curing time, and cavity design, were standardized to isolate the effect of composite composition on marginal adaptation. The differences observed in the marginal gaps between the groups can be attributed to material properties, such as the resin matrix, filler content, and polymerization characteristics. SDR Plus, a bulk-fill composite, exhibited better marginal adaptation than fiber-reinforced and nanofilled composites, likely because of its lower polymerization shrinkage (due to incremental application) and its ability to be placed in thicker layers with minimal stress on the tooth structure. These findings are consistent with studies showing that bulk-fill composites exhibit better marginal integrity owing to their optimized resin chemistry (modified monomer composition) and reduced polymerization stress [16].

Fiber-reinforced composites (EverX, which contain short glass fibers) exhibited the largest marginal gaps in this study. Short glass fibers (0.2-0.6 mm) are mixed with the resin content, and they are randomly oriented in the matrix. Although these materials are designed to enhance mechanical strength and fracture resistance, their fiber content may interfere with the flow and adaptation to cavity walls, especially in intricate or deep restorations. This finding is supported by previous research, where fiber-reinforced composites demonstrated poor marginal adaptation owing to the impact of the fibers on the polymerization stress distribution [17]. Although fiber-reinforced composites excel in terms of reinforcing weakened tooth structures, their performance in marginal adaptation appears to be compromised in the present study.

Nanofilled composites also demonstrated larger marginal gaps than bulk-fill composites. Although nanofilled composites are designed to offer improved aesthetics and polishing ability, their higher filler content can increase viscosity, thereby reducing their ability to adapt well to cavity walls. This result aligns with the findings of a study by AlSagob et al. [7,8], who reported that nanofilled composites, despite their excellent mechanical properties, can exhibit significant polymerization shrinkage and marginal gap formation when placed in deep or complex cavities, which may be due to the high resin-to-filler ratio and use of Bis-GMA monomer.

Comparing the results of this study with those in the literature, it is evident that bulk-fill composites, such as SDR Plus, generally provide better marginal adaptation than fiber-reinforced and nanofilled composites. A recent study by Fronza et al. [18] supports this observation, indicating that bulk-fill composites exhibit lower marginal gap formation owing to their lower shrinkage stress and better handling properties. In contrast, fiber-reinforced composites have shown limited success in achieving optimal marginal adaptation, as also noted by Garoushi et al. [17], who found that their rigid fiber structure impairs their ability to conform to cavity margins.

Limitations

This study included a relatively modest sample size, which restricts the applicability of the results to a broader population. Additionally, the exclusion criteria, such as the exclusion of teeth with caries, previous restorations, or visible fracture lines, could introduce selection bias and may not accurately represent the clinical scenario. Moreover, the in-vitro design of our study may not completely duplicate the complex oral environment as factors such as salivary flow, occlusal forces, and bacterial presence were not accounted for, which can potentially affect the performance of restorative materials differently in clinical settings. In this study, we employed a widely accepted aging technique, thermocycling, to replicate the gradual weakening of bonds within the oral environment over time, which is a topic of debate among scholars.

## Conclusions

Marginal adaptation with composite restoration is a primary requisite for the success of endodontic treatment. The present study showed that the bulk-fill flowable composite (SDR Plus) is better at marginal adaptation at the tooth-restoration interface than fiber-reinforced and nanohybrid composites. Conventional composites have a simple composition and a long history of reliable performance. Nanohybrid and fiber-reinforced composites, owing to their complex formulation and specific structural components, can be more technique-sensitive and less adaptable to cavity walls. Therefore, bulk-fill flowable (SDR plus) composites are recommended for the restoration of ETT.
